# Development of a Website for a Living Network Meta-analysis of Atopic Dermatitis Treatments Using a User-Centered Design: Multimethod Study

**DOI:** 10.2196/41201

**Published:** 2022-09-26

**Authors:** Karen P L Lau, Payal Agarwal, Laura Parente, Olivia Marcello, Mike Lovas, Jason Van, Simone N Vigod, Trevor Champagne, Tanya Mohan, Bernd WM Arents, Tim Burton, Carsten Flohr, Aaron M Drucker

**Affiliations:** 1 Women's College Research Institute Women's College Hospital Toronto, ON Canada; 2 Family and Community Medicine University of Toronto Toronto, ON Canada; 3 Healthcare Human Factors University Health Network Toronto, ON Canada; 4 Cancer Digital Intelligence Princess Margaret Cancer Centre University Health Network Toronto, ON Canada; 5 Thousand Plus Toronto, ON Canada; 6 Institute of Health Policy, Management, and Evaluation Dalla Lana School of Public Health University of Toronto Toronto, ON Canada; 7 Department of Psychiatry University of Toronto Toronto, ON Canada; 8 Dermatology Division Sunnybrook Health Sciences Centre Toronto, ON Canada; 9 Specialty Health Network Shoppers Drug Mart Toronto, ON Canada; 10 Dutch Association for People with Atopic Dermatitis Nijkerk Netherlands; 11 Unit for Population-Based Dermatology Research St John's Institute of Dermatology Guy's & St Thomas' NHS Foundation Trust and King's College London London United Kingdom; 12 Division of Dermatology Department of Medicine University of Toronto Toronto, ON Canada

**Keywords:** atopic dermatitis, skin, dermatology, dermatitis, eczema, network meta-analysis, knowledge translation, health literacy, user-centered design, patient education, information seeking, health information, website development, web development, web design, website design, allergist, user experience, interface, usability, visualization, decision-making, online resource, education material

## Abstract

**Background:**

A rapid expansion of systemic immunological treatment options for atopic dermatitis (AD) has created a need for clinically relevant and understandable comparative efficacy and safety information for patients and clinicians. Given the scarcity of head-to-head trials, network meta-analysis (NMA) is an alternative way to enable robust comparisons among treatment options; however, NMA results are often complex and difficult to directly implement in shared decision-making.

**Objective:**

The aim of this study is to develop a website that effectively presents the results of a living systematic review and NMA on AD treatments to patient and clinician users.

**Methods:**

We conducted a multimethod study using iterative feedback from adults with AD, adult caregivers of children with AD, dermatologists, and allergists within a user-centered design framework. We used questionnaires followed by workshops among patients and clinicians to develop and improve the website interface. Usability testing was done with a caregiver of a patient with eczema.

**Results:**

Questionnaires were completed by 31 adults with AD or caregivers and 94 clinicians. Patients and caregivers felt it was very important to know about new treatments (20/31, 65%). Clinicians felt the lack of evidence-based comparisons between treatments was a barrier to care (55/93, 59%). “Avoiding dangerous side effects” was ranked as the most important priority for patients (weighted ranking 5.2/7, with higher ranking being more important), and “improving patients’ overall symptoms” was the most important priority for clinicians (weighted ranking 5.0/6). A total of 4 patients and 7 clinicians participated in workshops; they appreciated visualizations of the NMA results and found the website valuable for comparing different treatments. The patients suggested changes to simplify the interface and clarify terminology related to comparative efficacy. The user in the usability testing found the website intuitive to navigate.

**Conclusions:**

We developed a website, “eczematherapies.com,” with a user-centered design approach. Visualizations of NMA results enable users to compare treatments as part of their shared decision-making process.

## Introduction

Progress in understanding the immunopathogenesis of atopic dermatitis (AD) has resulted in an expansion of systemic immunomodulatory treatments. A recent review found over 70 compounds being studied in clinical trials [1]. Expanded therapeutic options should improve outcomes for people with AD, but treatment decisions may become more complex. Comparing the relative effectiveness and safety of different medications is challenging because most clinical trials are placebo-controlled, with few head-to-head trials [2,3]. Network meta-analysis (NMA) can address this gap by using direct and indirect evidence to compare treatments with each other, including treatments that have never been compared with each other in a head-to-head trial [2,4]. We conduct a *living* systematic review and NMA of systemic immunomodulatory treatments for AD that is updated regularly to provide up-to-date comparative evidence [2,3].

Living NMAs have great potential to facilitate continuous knowledge synthesis across different fields of medicine, but the outputs of NMAs can be challenging to interpret for patients and clinician end users. There are resources on creating NMA network diagrams and forest plots for publication [4,5], but these are often complex and may not be clinically meaningful. Some groups have attempted to share NMA results using an open science approach by making their living NMAs available on websites [6-9], but these websites resemble traditional knowledge translation outputs such as journal publications and conference presentations; understanding the results is likely difficult for non–researcher knowledge users [10]. Clinicians and patients without training in interpreting NMA results would not likely be able to use this information directly for treatment decisions. Stakeholder engagement in the website design process could improve uptake and dissemination of NMA results [11].

Our overall goal is to provide reliable information on the relative efficacy and safety of systemic treatments for AD and to help inform clinical shared decision-making. The objective of this study was to develop a website to effectively present the results of our living systematic review and NMA of AD treatments to patients and clinicians.

## Methods

### Study Design and Setting

To design and develop the website, we used a multimethod user-centered approach. User-centered design has been shown to increase the overall adoption and impact of health tools [12]. We used best practices for user-centered design of decision aids, including a 3-phased iterative approach, with feedback from patients and clinicians [13]. Our team consisted of clinicians, a patient partner, digital product designers, and web developers. The development process took place between September 2019 and April 2020 in Toronto, Ontario, Canada. We completed the study in the following three phases: (1) patients and caregivers of patients with AD and clinicians who treat AD completed questionnaires about meaningful criteria for seeking evidence-based information regarding AD treatments; (2) two workshops, one with patients and caregivers and another with clinicians, assessed how participants perceived and wanted to see the NMA results on the web interface; and (3) usability testing with a caregiver was conducted to identify the remaining barriers and receive feedback about navigation and usability of the website.

### Ethics Approval

This study was approved by the Women’s College Hospital Research Ethics Board (REB# 2019-0095-E).

### Website Design

Two digital product designers worked with the study investigators to design a prototype with visualizations of the NMA results. We chose to use horizonal bar charts to display the effectiveness of each of the treatments within a specific priority type. The bars represent surface under the cumulative ranking curve (SUCRA) values, an NMA output used to rank treatments within a given outcome; higher values, to a maximum of 100%, indicate better efficacy [4]. The decision to use this type of graphic was made as it is a visualization understood by a wide audience and allows for a simple way of comparing complex data, where concrete numbers and percentages may have misrepresented the results of the NMA.

The different colors within the priority groups allow users to easily scan the page for that individual priority, and the white splitters within the bars act as visual markers to help users see how much of the bar is filled, without calling out a specific SUCRA percentage (because precise SUCRA point estimates oversimplify results).

The color fills on the bar charts are based on the data collected in the NMA, and therefore the lengths of the bars will only change when new data are analyzed and incorporated into the tool. The interactive component of this website comes into play when comparing one drug to another. Based on their first assessment of the represented graphics, users can select 2 medications they would like to compare side by side; they can view a table that, using written word and a large green checkmark, will clearly identify which of the 2 drugs is currently the most effective treatment option for a given priority, and help them decide which treatment may be better suited for them.

### Phase 1

Adults with AD and caregivers of children with AD were recruited from dermatology clinics at Women’s College Hospital. To be included, participants had to be 18 years or older and speak English. Consenting participants were given paper questionnaires to complete during clinic visits (Multimedia Appendix 1). At the end of the questionnaire, participants could opt in or out of being contacted about participating in the workshops.

A web-based questionnaire was circulated to allergists and dermatologists through the Canadian Dermatology Association and the Canadian Society of Allergy and Clinical Immunology mailing lists (Multimedia Appendix 1). Participation was anonymous.

### Phase 2

Workshops took place at Women’s College Hospital. Adults with AD and caregivers who indicated their interest in workshops on the Phase 1 questionnaires were recruited. Convenience sampling was used to recruit participants for the clinician workshop; email invitations were sent to dermatologists in the Toronto area.

Participants were shown a prototype of the website on a large television screen. Digital product designers navigated through various sections of the website to focus the discussion on the content, layout or hierarchy of information, and visualization of NMA results. Because of the different levels of familiarity with medical terminology between patients and clinicians, we decided to develop 2 separate web pages to tailor to each user group’s needs. The patient group shared their user experience and commented on the language on the home page, patient landing page, and 2 versions of the patient NMA results page. The clinician group was guided through the home page, clinician landing page, and research page. They shared their comments on the language and their expectations for each subsection.

The workshops were audio recorded. Two digital product designers took notes during the workshops and grouped the comments into high, medium, and low priority. High-priority items were those that were agreed upon by several participants and were perceived as valuable for improving website usability. Low-priority items were expressed by 1 or 2 participants and did not significantly affect how they used the website.

The designers and clinician researchers reviewed the suggestions and decided which priorities were critical or feasible to implement on a new version of the website.

### Phase 3

A caregiver of a patient with AD completed usability testing of the updated website, facilitated by 2 designers and 1 clinician (AMD). They reviewed the home page, “About Us” page, patient page (both results for children and for adults), and the experimental drugs page. The digital designers took notes and sorted the comments into high, medium, and low priority using the same criteria as the workshops. Additional information and revision of language were added to the final version of the website.

### Statistical Analysis

Descriptive statistics were used to summarize the questionnaire data. For ranking questions, the average ranking was calculated for each answer choice. Weights were applied in reverse; with the most preferred choice (ranked first) given the highest weight and the least preferred choice (ranked last) given the weight of 1. The answer choice with the highest average ranking is the most preferred choice.

## Results

### Patient Questionnaire Results

Questionnaires were completed by 31 adults with AD or caregivers (Table 1). Of these, 22 (71%) participants indicated they or their child have been on or have considered using systemic medications. Most participants (20/31, 65%) felt it was very important to know about new treatment options with a 10/10 rating. Most participants learned about new treatments from their doctor (29/31, 98%).

Participants felt effectiveness and side effects were very important information when learning about a new treatment. Other considerations when deciding on a new treatment include cost or insurance coverage, convenience, and length of treatment. “Avoiding potentially dangerous side effects” (5.2/7 weighted ranking; higher ranking indicates higher importance) and “improvement in quality of life” (4.9/7) were ranked the most important considerations.

When asked what they would do next with information about a new treatment option that aligns with their needs, most participants responded they would speak with their doctor. Most participants (16/31, 52%) were interested in knowing about drugs that are only available in countries outside of Canada.

**Table 1:** Demographics of patient questionnaire respondents (n=31).

Characteristics		Values, n (%)	
**Age range**			
	18-39		22 (71)
	>40		9 (29)
**Sex**			
	Female		18 (58)
	Male		13 (42)

### Clinician Questionnaire Results

Clinician questionnaires were completed by 94 participants (Table 2). Most (85/94, 90%) clinicians were seeing patients with AD at their practice. Many clinicians (55/93, 59%) felt the lack of evidence-based comparisons between treatment options was a barrier to patient care.

Clinicians ranked improvement in patients’ symptoms (5.0/6 weighted ranking; higher ranking indicated higher importance) and quality of life (4.0/6) as the highest priorities when deciding on a treatment. Other considerations when treating AD include age of patient, patient preference, and ease of use. They believed that efficacy, safety, and cost were the most important factors for their patients.

Most clinicians (60/90, 67%) indicated they would tell their patients about treatments that are not yet approved with the purpose of potentially enrolling patients into available trials or to give them hope. When asked where they are currently accessing research about treatment options, most clinicians mentioned journal articles and academic meetings as their primary sources of information.

**Table 2:** Demographics of clinician questionnaire respondents (n=94).

Characteristics		Values, n (%)
**Age range**		
	18-39	42 (45)
	40-59	31 (33)
	>60	21 (22)
**Sex^a^**		
	Female	55 (59)
	Male	38 (40)
**Number of years in practice**		
	Still in residency	7 (8)
	Less than 5 years	28 (30)
	6-10 years	11 (12)
	11-20 years	20 (22)
	21-30 years	8 (9)
	>30 years	19 (20)
**Type of practice**		
	Community	44 (47)
	Academic	17 (18)
	Community and academic	33 (35)

^a^Participants can choose not to say as a response to this question.

### Patient Workshop Results

A total of 4 participants (mean age 39 [SD 21.28] years; 2/4, 50% female; mean age at AD diagnosis: 19 [SD 28.58] years) participated in the patient workshop. They had previously tried a range of topical, phototherapy, and systemic treatments. Participants had a range of educational attainment from high school to professional or graduate degrees.

Two digital product designers guided the participants through several sections of the prototype with a focus on their understanding of the various outcome domains (eg, improvement in itch, improvement in quality of life, avoiding potentially dangerous side effects, etc) for each drug and the visualization of the NMA results. Overall, their feedback was positive; they felt it presented reliable information that gave them hope that more treatments were in the pipeline. They understood the goal of the website and stated that its affiliation with a teaching hospital and listed researchers gave the website more credibility. A high priority for the participants was the ability to see all the results at once without having to preselect individual outcome domains.

**Figure 1:**
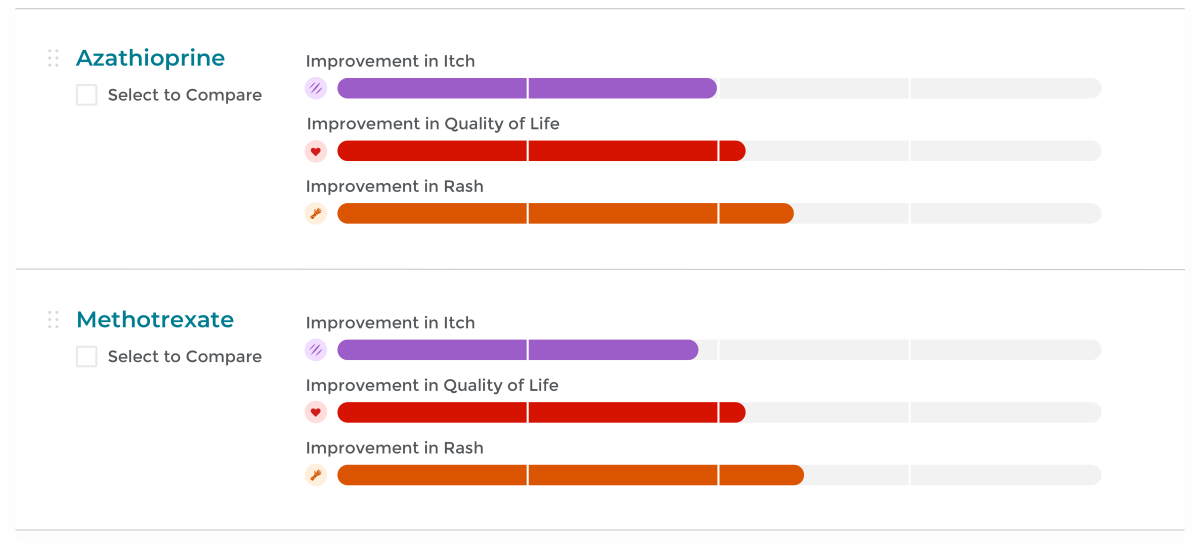
Visualization of network meta-analysis results from the patient website page. The colored bars represent effectiveness on various outcome domains (ie, itch, quality of life, improvement in rash). Users can also select 2 medications for a more detailed head-to-head comparison.

Participants had difficulty understanding the meaning of “relative effectiveness” and why each result was linked with a “certainty rating” (based on Grading of Recommendations, Assessment, Development and Evaluations [GRADE]) [14]. Based on their feedback, we changed the wording of “relative effectiveness” to “how do these drugs compare?”. We simplified the workflow of the website so NMA results would be displayed with fewer clicks. We also removed several outcome domains and the certainty information from the patient page (Figure 1).

### Clinician Workshop Results

A total of 7 clinicians (mean age 36 [SD 6.05] years; 4/7, 57% female; mean 6 years in independent practice) who treat patients with AD participated in the workshop. Clinicians were all dermatologists working in either academic or community group or solo practices. They reported seeing between 2 and 10 AD patients per month.

They understood both “relative effectiveness” and GRADE certainty information. Similar to patients, they wanted to see all the results at once with as few clicks as possible. They felt reassured that the website clearly states it is not affiliated with pharmaceutical companies. A medium-level priority for them was a request for a drug information card when they clicked on the name of each drug. Overall, they understood the presented results but were uncertain whether the information would be clinically meaningful in their practice because at the time of the workshop there was only one targeted medication approved for AD. They felt it was an easy-to-use resource if they wanted to learn more about new treatments.

### Usability Testing Results

A caregiver of a child with AD participated in a remote usability testing session with 2 digital product designers and 1 clinician investigator (AMD). The user’s expectation from the home page was that she would learn more information about eczema research and upcoming clinical trials. She did not have any issues navigating the website and had no difficulty understanding its content. She believed the longer bars on the “Avoiding potentially dangerous side effects” domain meant more dangerous side effects. The wording was then changed to “Safety: Fewer Serious Adverse Events.”

## Discussion

### Principal Findings

We created a knowledge translation website for a living network meta-analysis of AD treatments, employing a user-centered design approach and iterative feedback from patients, caregivers, and clinicians. The website [15] was launched in April 2020, and since then, we have posted 6 NMA result updates. According to our website analytics (assessed June 13, 2022), it has been visited 7418 times by users from over 65 countries. There were 887 active users over the previous 30 days, suggesting it has enduring utility.

Our questionnaire found that learning about new AD treatments is a high priority for adults with AD and caregivers of children with AD. Most of the participants expected to learn this information from their physicians, so it is important to disseminate new treatment information to clinicians treating AD. Clinicians were motivated to tell their patients about not-yet-approved treatment options, but many felt that the lack of evidence-based comparisons between treatments can impede care. There was an apparent need among patients and clinicians for a tool that can help them better understand and compare new AD treatment options.

In workshops, we received overall positive feedback about the website from participants who provided suggestions to improve the usability of the website. Their insights on data visualizations and language contributed to the subsequent interface design. Patients and clinicians were satisfied with similar data visualizations, with some simplification on the patient page. Usability testing with a caregiver found that the final design was easy to navigate and understand.

Our website achieves the following 2 goals of knowledge translation for our NMA results: (1) open science, in which information is disseminated in an available, transparent, and timely manner; and (2) dissemination of useful information to end users (ie, patients and clinicians). Researchers usually rely on passive knowledge translation strategies such as journal publications and conference presentations [16]. Passive knowledge translation approaches are less likely than active knowledge translation approaches to result in uptake of the information and often lack stakeholder engagement. An active knowledge translation approach that involves end users in the development process may lead to better uptake [17].

Other living NMA websites achieve the open science goal of disseminating NMA results. The COVID-NMA Initiative group has developed a living mapping and systematic review of COVID-19 trials [6,7]. Users can use its interface to perform their own meta-analyses using the COVID-NMA’s frequently updated database. Similar living NMA websites have developed sophisticated interactive data visualizations, but users without training in NMA methodology may find it difficult to interpret the results [8,9]. Compared to other living NMA websites, our research page is less sophisticated and interactive. Living NMA websites are an improvement over traditional knowledge translation strategies in that they are more efficient at delivering up-to-date information to other researchers, but dissemination and uptake need to reach clinicians and patients in order bridge the gap between science and practice.

Our website was specifically designed to disseminate NMA results to end users, with specific pages dedicated to researchers, patients, and clinicians. Similar to other living NMA websites, our research page posts extensive data from our NMA results. The patient and clinician pages display the NMA results using easy-to-understand comparative visualizations.

### Limitations and Future Directions

For feasibility, patient and caregiver participants were recruited from a single urban tertiary care center in Canada. Clinician survey participants were recruited only from Canada, and clinician workshop participants all worked in the Toronto area. We only conducted final usability testing with a single end user; however, our research team included clinicians and patients who also provided iterative feedback as the website was in development. Our findings may not be fully generalizable to all end users; additional testing with more users on the final website product would be informative.

One of the aims of the website is to provide a treatment comparison tool for patients with AD and clinicians. A user experience study can investigate users’ purpose for the website and whether their goals align with those we set out. To further improve user experience, it may be worthwhile to add a short video with an introduction to the website and a basic overview of NMA methodology. Research has found that videos are an effective knowledge translation tool and can lead to overall knowledge improvement [18].

Traditionally, research impact is measured by bibliometric measures such as Impact Factor and citation counts [19]. As open science expands to wider, nonacademic audiences, it may be worthwhile to consider alternative metrics (*altmetrics*) to better capture other forms of dissemination that are more accessible and popular among nonacademic knowledge users [17]. Altmetrics can assess dissemination of research to groups outside the scientific community by aggregating mentions in media outlets such as blogs, forums, discussion sites, and social media such as Twitter and Facebook [17].

### Conclusions

To address the need among patients and clinicians for evidence-based information on systemic AD treatments, we developed a website to present results from a living systematic review and NMA. Engaging end users during the design and development process resulted in a tool that makes complex NMA results more relevant to their treatment decision-making process.

## Appendix

Multimedia Appendix 1 Questionnaires.

## Abbreviations

AD: atopic dermatitis
GRADE: Grading of Recommendations, Assessment, Development and Evaluations
NMA: network meta-analysis
SUCRA: surface under the cumulative ranking curve
