# Supplementary material for: Development of a Website for a Living Network Meta-analysis of Atopic Dermatitis Treatments Using a User-Centered Design: Multimethod Study
Source: JMIR Dermatol. 2022 Sep 26;5(3):e41201. doi: 10.2196/41201 (PMC10334921; doi:10.2196/41201)
Supplement: Multimedia Appendix 1 [file derma_v5i3e41201_app1.docx]

## Multimedia Appendix 1

Patient questionnaire

| Questions and answer choices | n (%) |
| --- | --- |
| *Which systemic treatment have you tried?* |  |
| Pills  Injections  Others | 2 (6%)  16 (52%)  15 (48%) |
| *How important is it to you to know about new treatment options? (circle one) scale: 1 - 10 (1 = unimportant; 10 = very important)* | Mean (SD) |
|  | 9.19 (1.38) |
| *The last time you learnt about treatment options for your eczema, where did you get the information?* |  |
| Doctor  Pharmacist  Online  Other | 29 (98%)  0  2 (6%)  1 (3%) |
| *Is knowing about drugs that are currently in clinical trials a priority for you? (E.g., drugs that are in clinical trials may only be available in 5 years)* |  |
| Yes  No | 14 (45%)  17 (55%) |
| *Are you interested in knowing what drugs are available for treatment in other countries?* |  |
| Yes  No | 16 (52%)  15 (48%) |

Patient ranking question: What below is most important to you when deciding on a treatment? (Rank from 1 to 7 with 1 being the most important)

Answered: 31 Incorrectly filled: 8

| Domain | Average ranking score /7 |
| --- | --- |
| Improvement in itch | 4.6 |
| Improvement in overall symptoms, including dryness and crusting | 4.5 |
| Improvement in quality of life | 4.9 |
| Improvement in visibility of rash | 3.2 |
| Avoiding potentially dangerous side effects | 5.2 |
| Avoiding any side effects that would make you feel like stopping the treatment | 3.8 |
| Cost of treatment/insurance coverage | 3.3 |

Clinician questionnaire

| Questions and answer choices | n (%) |
| --- | --- |
| *Do you consider the lack of evidence-based comparisons between*  *different treatment options for atopic dermatitis to be a barrier in the care*  *of your patients?* |  |
| Yes  No | 55 (59%)  38 (41) |
| *Are you currently treating patients with atopic dermatitis who are*  *either considering systemic treatments or have used systemic treatments*  *in the past?* |  |
| Yes  No | 85 (90%)  9 (10%) |
| *When assessing a patient with atopic dermatitis, would you*  *discuss/educate your patients on treatments that are currently in clinical*  *trials (i.e not yet approved by Heath Canada)?* |  |
| Yes  No | 60 (67%)  30 (33%) |

Clinician ranking question: Which of the following is most important to you when deciding on a

treatment? (Rank below from 1 to 6 with 1 being the most important)

| Domain | Average ranking score /6 |
| --- | --- |
| Patient Symptoms (how substantial the expected improvement in patients’ symptoms is, such as itch and sleep loss) | 5.0 |
| Improvement in Rash (how substantial the expected improvement in the patients’ visible rash is) | 3.4 |
| Improvement in quality of life | 4.0 |
| Cost of Treatment/ Insurance Coverage | 2.8 |
| Safety of Treatment (how often serious adverse events occur while patients take these drugs) | 3.9 |
| Avoiding any side effects that would make your patient feel like stopping the treatment | 2.2 |
